# 急性早幼粒细胞白血病的治愈之路与中国贡献

**DOI:** 10.3760/cma.j.cn121090-20250307-00119

**Published:** 2025-05

**Authors:** 丽 陈, 赛娟 陈

**Affiliations:** 上海血液学研究所，组学与疾病全国重点实验室，国家转化医学研究中心，上海交通大学医学院附属瑞金医院血液科，上海 200025 Shanghai Institute of Hematology, State Key Laboratory of Medical Genomics, National Research Centre for Translational Medicine at Shanghai, Department of Hematology, Ruijin Hospital Affiliated to Shanghai Jiao Tong University School of Medicine, Shanghai 200025, China

**Keywords:** 白血病，早幼粒细胞，急性, 全反式维甲酸, 三氧化二砷, 协同靶向治疗, Leukemia, promyelocytic, acute, All-trans retinoic acid, Arsenic trioxide, Synergistic targeted therapy

## Abstract

急性早幼粒细胞白血病（APL）曾因早期高死亡率被称为“最凶险的白血病”，其治疗变革是肿瘤协同靶向治疗的里程碑。全反式维甲酸（ATRA）和三氧化二砷（ATO）的发现与应用，使APL的5年总生存率从不足35％提升至90％以上。中国血液肿瘤学界在此过程中作出重要贡献：王振义教授研究组开创ATRA分化疗法，张亭栋教授研究组验证ATO的临床价值。上海血液学研究所团队克隆了APL特异的15和17号染色体易位形成的PML::RARA融合基因，并发现了首个PLZF::RARA变异型融合基因，在阐明APL分子细胞发病机制和有效药物作用机制基础上，提出了ATRA和ATO协同靶向治疗初发APL的方案，在多中心临床试验中取得了95.7％的7年无复发生存率。之后，国内外数个团队又开发了费效比高的口服砷剂（复方黄黛片或口服ATO溶液）与ATRA的联合疗法，适于在资源有限地区推广。本文系统回顾APL治疗的关键突破，解析科学机制与临床意义，并展望未来机遇和挑战。

## Abstract (en)

Acute promyelocytic leukemia（APL）was historically regarded as the most aggressive subtype of acute leukemia due to its high early mortality rate. The transformation in APL treatment represents a milestone in targeted cancer therapy. The discovery and clinical application of all-trans retinoic acid（ATRA）and arsenic trioxide（ATO）have dramatically improved the prognosis of APL, increasing the 5-year overall survival rate from less than 35% to over 90%. Chinese hematology/oncology community have made significant contributions to this transformation: Professor Wang Zhenyi's group pioneered ATRA-induced differentiation therapy, while Professor Zhang Tingdong's group verified the clinical efficacy of ATO. The research team of Shanghai Institute of Hematology（SIH）cloned the PML::RARA fusion gene resulting from the chromosomal translocation（15;17）and the first variant PLZF::RARA fusion gene. Based on the characterization of the leukogenesis at molecular and cellular levels and the mechanisms of action of effective drugs, the SIH team established a synergistic targeted therapy protocol for newly diagnosed APL patients, achieving a disease-free survival rate of 95.7% in a nationwide multi-center clinical trial. Subsequently, several teams explored the use of oral arsenic（Realgar-Indigo naturalis formula or oral ATO solution）combined with ATRA to treat APL, which is of high cost-effectiveness and can be promoted in resource-restricted regions. This review systematically summarizes the key therapeutic breakthroughs in APL, elucidates the underlying scientific mechanisms and clinical significance, and identifies remaining challenges for optimizing disease management.

急性早幼粒细胞白血病（APL）占急性髓系白血病（AML）的10%～15%，以15号和17号染色体易位形成的PML::RARA融合基因为特征，干扰正常维甲酸信号通路，导致髓系细胞分化阻断于早幼粒细胞阶段，并伴有严重凝血功能障碍。20世纪80年代前，APL早期死亡率高达30%～50%，中位生存期不足1年[1]–[2]。过去四十年来，APL的治疗取得了革命性进展，全反式维甲酸（ATRA）和三氧化二砷（ATO）的成功应用，使APL成为首个通过协同靶向治疗实现高治愈率的恶性肿瘤。中国学者在APL的转化研究和精准诊疗中发挥了重要作用，推动了治疗模式从“强化化疗”向“精准、减毒、无化疗”转变[1]–[3]。本文系统回顾APL治疗策略的演变、优化过程及中国学者的贡献。

一、APL治疗的历史性变革

APL的治疗策略经历了四个主要阶段：化疗时代、ATRA联合化疗、砷剂时代，以及ATRA联合砷剂的靶向治愈模式。

1. 化疗主导的时代：1973年，Bernard等[4]发现蒽环类药物对APL细胞高度敏感，化疗可使75%～80%的患者获得完全缓解（CR），成为APL治疗的首次突破。然而，化疗加重凝血异常，增加早期死亡风险，且难以根除白血病干细胞，5年无病生存（DFS）率仅为35%～45%[1]。

2. ATRA的发现：对白血病细胞生物学特征的深入研究促成了分化治疗的概念。20世纪80年代初，Breitman等[5]–[6]发现13-顺式维甲酸（13-cis RA）可诱导白血病细胞分化。1985年，王振义教授领导的上海血液学研究所（上海血研所）团队发现RA的异构体ATRA疗效最佳，首批24例患者中23例获CR，外周血和骨髓中发现终末分化的分叶核中性粒细胞中带有白血病细胞特异的奥氏小体，奠定了APL治疗的里程碑。ATRA可迅速缓解凝血障碍，降低早期死亡率，同时不引起骨髓抑制和感染[7]。这一突破确立了“分化治疗”理念。在APL发病原理和维甲酸治疗机制方面，上海血研所陈竺、陈赛娟教授团队于1991年独立克隆了PML::RARA，解析APL发病机制[8]–[9]，1993年又在国际上首先报道了APL变异型染色体易位t（11;17），并克隆了11号染色体上的早幼粒锌指蛋白基因（PLZF）与RARA形成的PLZF::RARA融合基因[10]–[11]，建成了PML::RARA和PLZF::RARA转基因小鼠模型，证明两种融合基因均为APL的驱动基因，并发现PML::RARA融合蛋白在生理性维甲酸浓度（10^−9^ mol/L）下，可与核受体共抑制物结合，抑制靶基因表达而阻断细胞分化，药理浓度维甲酸（10^−6^ mol/L）作用则可使共抑制物解离而募集共激活物从而启动下游基因表达和APL细胞的终末分化；而PLZF::RARA即使在药理浓度维甲酸作用下仍不能解除与共抑制物结合，从而导致对ATRA耐药，为阐明ATRA诱导分化治疗APL的分子机制奠定了基础[12]–[14]，表明ATRA对APL的作用本质上是一种分子靶向治疗。此外，该团队还克隆了APL细胞分化过程中受ATRA调控的一系列基因（RIG），并对其进行了结构和功能研究，证明了若干RIG基因是参与髓系祖细胞分化调控的关键基因[15]–[17]。近期，上海血研所王侃侃、陈竺教授团队的研究揭示，PML::RARA不仅发挥经典的转录抑制作用，还通过超级增强子（super-enhancer）介导的基因激活功能促进白血病发生发展，拓展了对致癌融合蛋白作用机制的理解，为未来精准治疗提供了新思路[18]。

3. ATRA联合化疗：ATRA单药治疗易耐药，复发率高，多数患者在CR后6个月内复发，主要原因为ATRA的分解代谢增强以及PML::RARA中RARA部分的配体结合区域发生突变[7],[19]–[20]。20世纪90年代初期，中国开展的大规模临床研究证实，ATRA联合化疗可显著降低复发风险[21]。此外，全球多项前瞻性研究表明，ATRA联合蒽环类化疗可使一半至三分之二的患者达到长期缓解，因此成为APL一线治疗标准[22]–[24]。

4. 砷剂的临床转化：1992年，哈尔滨医科大学附属第一医院（哈医大附一院）张亭栋教授团队首次报道含砷制剂“癌灵1号”（主要成分为ATO和汞）治疗初治APL，65%患者获得CR[25]。该院张鹏教授团队随后证实，ATO对初治和复发难治APL患者均有效，CR率分别达到89%和52%。1995年，上海血研所联合哈医大附一院开展的临床试验进一步显示，单纯的ATO（亚砷酸注射液）治疗复发APL疗效显著，复发患者CR率达85%～90%[26]–[28]，但再次缓解后绝大多数又于短期内复发。在该研究中还注意到ATRA与ATO联合用药者的疗效较单用ATO者更好[28]。此外，该团队发现ATO单药治疗可使70%的初发患者获得较长期的分子学缓解（CMR），该结果为国际上数个研究所证实[29]–[32]。上海血研所提供的临床试验结果上报国家药监部门后，使亚砷酸注射液最终于1999年批准作为新药上市。机制研究表明，ATO具有剂量依赖性双重作用：高浓度（0.5～2.0 µmol/L）时诱导细胞凋亡，低浓度（0.1～0.5 µmol/L）时促进细胞分化[33]，其作用主要通过靶向PML结构域，在转录后修饰和蛋白水平降解PML::RARA并诱导细胞凋亡[33]–[36]。此外，ATO可有效清除APL中的白血病干细胞，这可能是ATO单药能够实现长期缓解的关键机制[37]。

5. ATRA联合ATO：上海血研所团队深入研究后发现，ATRA和ATO的作用靶点均为APL的致病蛋白PML::RARA，但两者作用途径不同：ATRA通过调变异常核受体RARA介导的转录抑制或激活功能而发挥作用，亦可通过较长时间作用后引起融合蛋白轻度降解；ATO则与PML::RARA或PML的环指-B盒-螺旋环螺旋（RBCC）结构域中的RING和B-Box2模体中的半胱氨酸巯基结合，诱导蛋白构象变化，诱导PML发生SUMO-泛素化修饰，导致PML::RARA在溶蛋白小体的迅速降解[36]。ATRA和ATO联合作用APL细胞后，PML::RARA的降解明显加快[38]。ATRA与ATO在APL细胞模型和动物实验中也表现出协同作用，可共同诱导APL细胞分化凋亡，清除白血病起始细胞[38]–[40]。以上述工作为基础，1999年，上海血研所提出了将ATRA和ATO联合应用于初发APL治疗有可能根治该型白血病的科学假设，并于2000年开始了临床试验。研究表明，采用ATRA联合ATO为基础的方案可显著提高APL的疗效。该方案分为三个治疗阶段：诱导治疗使用ATRA+ATO+伊达比星（IDA）三药联合，巩固治疗依次应用DA方案［柔红霉素（DNR）+阿糖胞苷（Ara-C）］，中剂量Ara-C以及HA方案［高三尖杉酯碱（HHT）+Ara-C］，维持期则采用ATRA/ATO/6巯基嘌呤（6-MP）/甲氨蝶呤（MTX）序贯治疗。与单用ATRA或ATO的诱导方案相比，该联合治疗方案展现出显著优势：CR率高达94.1%，获得CR所需时间明显缩短，诱导缓解和巩固治疗后PML::RARA转录本清除幅度更大，且18个月中位随访期的DFS率显著提高[24]。在后续对85例APL患者的随访中证明，5年总生存（OS）率达91.7%，CR患者的5年无复发生存（RFS）率高达94.8%[41]，且长期随访无明显不良反应[42]。这些结果发表后被国际同行称为“上海方案”[43]，被国际指南采纳，奠定了APL的ATRA+ATO靶向治愈模式。

二、APL治疗的优化探索

APL的优化治疗目标是在维持高疗效的同时，减少化疗相关毒性，提高患者长期生存率和生活质量。这一进程可概括为从“强化化疗”向“精准减毒”发展。

1. 风险分层治疗：2000年，西班牙Sanz教授提出基于外周血WBC和PLT的APL风险分层体系（Sanz分层），将APL患者分为低危（WBC≤10×10⁹/L，PLT>40×10⁹/L）、中危（WBC≤10×10⁹/L，PLT≤40×10⁹/L）和高危（WBC>10×10⁹/L）三组[44]。这一分层体系为个体化治疗提供了依据，低危患者可减少化疗以降低毒性，而高危患者需强化治疗以提高长期生存率。

2. 化疗时代的优化探索：在风险分层的指导下，研究者针对不同风险患者优化化疗方案，以提高疗效并降低复发率。西班牙PETHENA小组在LPA99研究中首次应用Sanz分层，在LPA96研究的基础上优化中高危患者的巩固治疗，包括加入ATRA并提高IDA剂量，使3年累计复发率（CIR）从20%降至10%，但高危患者的复发风险仍然较高[45]。随后，LPA2005研究在LPA99基础上进一步优化，在高危患者的巩固治疗中加入中剂量Ara-C，使3年CIR由26%降至11%，而低中危患者在减少蒽环类化疗剂量的情况下仍能保持长期疗效[46]。类似地，意大利GIMEMA主导的AIDA2000研究在AIDA0493方案基础上，在巩固治疗中加入ATRA，并保留高危患者的中剂量Ara-C，使整体6年CIR由28%降至11%，高危患者的复发率也从50%降至10%。然而，尽管强化化疗降低了复发率，但其导致的骨髓抑制、感染及远期毒性问题日益突出，促使研究者探索减少甚至去除化疗的策略[47]。

3. 无化疗策略的崛起：随着ATO疗效的确立，研究者逐步验证其减少甚至完全替代化疗的可行性。澳大利亚APML4研究显示，诱导期加用ATO，巩固期以ATO替代化疗，可显著改善长期预后，并克服FLT3突变的不利影响[48]–[49]。美国MD安德森癌症中心（MDACC）进一步验证ATRA+ATO全程无化疗方案，仅在诱导期使用CD33单抗（吉妥珠单抗）或IDA控制高白细胞，5年无事件生存（EFS）率和OS率均超过85%[50]。欧洲APL0406研究进一步证实，在低中危APL患者中，ATRA+ATO无化疗方案不仅优于传统AIDA方案，还显著降低3～4级血液学不良反应的发生率，提高患者生活质量[51]–[52]。在此基础上，APOLLO研究进一步评估该方案在高危患者中的适用性，结果显示，ATO联合ATRA+短程IDA诱导可使5年OS率达93%，确立其为高危APL的新标准方案[53]。此外，两项大样本回顾性研究进一步证实，含ATO方案较不含ATO方案可显著改善预后，巩固了ATO在APL一线治疗中的核心地位[54]–[55]。

三、中国学者的优化实践

临床研究显示，ATRA联合ATO两药协同靶向治疗，7年DFS率达95%以上，而且长期安全性得到充分证实。

1. ATO在APL治疗中的优化应用：2012年，在陈竺和陈赛娟教授的指导下，上海血研所/瑞金医院血液科牵头开展了迄今为止国际上规模最大（855例）的初发APL多中心随机对照研究（APL2012研究），在“上海方案”的基础上，评估低危患者ATO是否可以完全替代化疗，以及中高危患者ATO是否可以减少化疗。结果显示，方案优化后，所有患者7年DFS率均超过90%，且ATO组与化疗组疗效相当（7年DFS率95.7%对92.6%）。此外，ATO组复发率更低（7年CIR 2.2%对6.1%，*P*=0.011），3～4级血液学毒性显著减少，安全性更优。该方案在维持高疗效的同时，明显减少了不良事件[56]，从而确立了ATO在巩固治疗阶段替代化疗的可行性，并为进一步优化APL治疗提供了循证依据。基于APL2012研究，上海瑞金医院血液科自2018年起在新诊断APL患者中全面采用ATO方案，并进一步优化策略，包括维持治疗期使用口服复方黄黛片（RIF）替代静脉ATO，以及高危患者巩固治疗期去除化疗。研究表明，巩固治疗期去化疗后，高危患者的长期DFS率和OS率未受影响，进一步确立了无化疗策略在APL中的应用价值[57]。此外，西安交通大学APL15研究同样验证了无化疗方案的可行性。结果显示，无论是低中危还是高危患者，ATRA+ATO方案与ATRA+ATO+化疗方案的长期生存率相当，证实即便在高危患者中，无化疗方案亦具备可行性[58]。需要注意的是，无化疗策略主要适用于诱导缓解的患者，而对于诱导治疗期WBC接近或超过10×10⁹/L的患者，不论风险分层如何，仍应及时使用蒽环类化疗药物控制白细胞升高，而不能仅依赖羟基脲[59]。

2. 口服砷剂的创新与临床转化：尽管砷是一种已知毒物，但它在中西医中均有悠久的药用历史。近年来，中国学者在口服砷剂的研究中取得了重要突破。

（1）雄黄（化学名：四硫化四砷，As_4_S_4_）：雄黄是一种砷化合物，早在2002年，北京医科大学血研所陆道培教授团队就报道，纯As_4_S_4_单药治疗初治和复发APL，所有患者都获得CR，CMR率达80%[60]。

（2）RIF：RIF（曾用名：复方青黛片）由As_4_S_4_、青黛、丹参和太子参四味药物组成。20世纪80年代，解放军第210医院黄世林教授团队在60例APL患者中首次报道了RIF的疗效，CR率达98%。随后的中国多中心研究不仅验证了这一结果，而且证实RIF耐受性良好[61]–[62]。上海血研所团队从分子水平阐明中药RIF治疗APL的“君、臣、佐、使”的配伍原则，发现As_4_S_4_可诱导PML::RARA快速降解，起“君药”作用；丹参酮可促进细胞分化相关基因的表达，可视为“臣药”；青黛的有效成分靛玉红具有阻断细胞周期的作用，是为“佐药”；而丹参酮与靛玉红通过上调负责运输砷的水孔通道蛋白9的含量，促使进入白血病细胞的砷明显增多，均起到“使药”的作用[63]。该研究为中医药学的现代化、国际化和中西医结合研究起到了示范作用。该药于2009年获中国国家药监局批准，正式用于APL治疗。

2000年起，北京大学血研所黄晓军教授团队系统研究RIF替代静脉ATO的可行性，推动APL砷剂治疗从静脉用药到口服模式转变[3]。2007年，该团队开展APL07多中心随机对照研究，评估RIF在诱导和维持治疗中的疗效。结果显示，RIF组CR率达99%，2年DFS率为98%，与静脉ATO组相当[64]。7年随访表明，两组EFS、OS和CIR差异无统计学意义[65]，确立了RIF的非劣效性。在此基础上，团队进一步评估RIF+ATRA无化疗方案在非高危APL患者中的应用。患者随机分为RIF组和ATO组，均联合ATRA诱导至CR。缓解后，予以ATRA（2周给药，2周停药）共7个周期，RIF或ATO（4周给药，4周停药）共4个周期。结果显示，两组2年EFS率（97%对94%）及OS率（100%对94%）差异无统计学意义[66]。随后，该方案应用于20例高危患者，所有患者均获CR，3年OS率100%，EFS率89.4%[67]，证实RIF+ATRA同样适用于高危患者，为未来无化疗策略提供重要依据。此外，无化疗门诊口服治疗模式降低了治疗成本，使RIF在中国APL治疗中更具吸引力[68]。

（3）口服ATO溶液：1998年，中国香港大学研究团队研制出1 mg/ml口服ATO溶液[69]，后续研究证实，其在APL一线和复发治疗中均具备良好疗效。在一项针对73例复发APL患者的长期随访研究中，患者接受DNR、口服ATO、ATRA及维生素C（AAA方案）诱导治疗，随后进行2个疗程巩固化疗和口服AAA维持治疗。最终，CR_2_患者5年OS率达79.5%，且无需自体或异基因造血干细胞移植[69]–[70]，表明既往接受过砷剂治疗的复发患者，使用口服ATO依然有效。2001至2013年间，口服AAA方案被用于CR_1_患者的维持治疗，替代传统ATRA联合口服化疗（6-MP+MTX），5年RFS率和OS率分别为90%和97%[71]。2013年起，该方案被纳入新诊断APL患者的一线诱导和维持治疗，并在高龄或合并症患者中去除DNR。结果表明，诱导治疗中加入口服ATO可显著降低复发风险，5年无白血病生存（LFS）率为100%，而未使用口服ATO诱导的患者为90.5%（*P*=0.03）。此外，APL一线治疗中口服AAA联合化疗，短期和长期心脏安全性良好，未观察到QTc间期延长、室性心律失常或心力衰竭的发生[72]。

（4）国外口服ATO制剂：澳大利亚研究团队开发了一种新型口服ATO胶囊，并在APML5临床研究中证实其与静脉ATO具有生物等效性，且安全性表现相似，同时口服ATO胶囊还显著降低了每日治疗费用[73]。此外，还有一种名为ORH-2014的口服ATO制剂，在美国MDACC开展的Ⅰ期临床试验中，ORH-2014（15 mg）与标准剂量静脉ATO（0.15 mg/kg）在生物利用度上具有一致性[74]。然而该制剂在APL领域的临床开发于2023年被暂停，其后续发展尚不明确[73]。

综上所述，口服砷剂在APL治疗中的应用不断扩展，从RIF到口服ATO溶液，再到新型口服ATO胶囊，为APL患者提供了更多便捷、高效且耐受性良好的治疗选择，推动了APL治疗从静脉输注向口服治疗的转变。

3. 可测量残留病（MRD）检测方法的优化与临床价值：1992年，上海血研所陈赛娟团队成为国际上首先建立RT-PCR方法用以检测APL细胞中PML::RARA转录本的团队之一[75]。之后，APL MRD的监测依赖于PML::RARA转录本的荧光定量PCR（qPCR）动态检测，其中，巩固治疗结束后的骨髓分子学检测对评估复发风险至关重要，实现CMR（MRD阴性）是巩固治疗的核心目标[76]。然而，随着ATRA联合ATO方案的广泛应用，APL患者的长期生存率显著提高，尤其是非高危患者，复发风险大幅降低[54]。因此，严格且长期的MRD监测可能带来的临床益处有限。

2019年欧洲白血病网络（ELN）指南建议，MRD监测应常规用于高危患者，而对于巩固治疗后已达到MRD阴性的非高危患者，无论其接受ATRA+ATO还是ATRA+化疗，都可以停止MRD监测。由于骨髓检测相较外周血能更早发现分子学复发，因此仍是MRD监测的首选方法[77]。但考虑到外周血检测的便捷性和创伤性小，且较血常规能更早识别分子学复发，通过提高检测频率，可在一定程度上弥补灵敏度的不足[78]。这一优化策略有助于在确保患者安全的同时，减少不必要的医疗资源消耗。

四、APL治疗的现存挑战和突破方向

尽管APL的治疗取得了显著进展，但仍存在挑战，主要包括早期死亡和复发。其中，初诊时WBC超过100×10^9^/L的超高危患者，出血性早期死亡风险极高，亟需有效的预防和治疗策略。

对于多次复发或对砷剂及维甲酸耐药的患者，治疗选择有限，新型靶向药物的开发为此类患者带来了希望。例如，CD33单抗通过靶向APL细胞表面的CD33抗原，增强ATO诱导的细胞毒性，已在初治和复发APL患者中显示出良好疗效[79]–[80]，但尚未在国内获批上市。此外，BCL-2抑制剂维奈克拉已在多种复发难治血液肿瘤中改善缓解和延长生存。研究显示，APL细胞高表达BCL-2并依赖氧化磷酸化，为维奈克拉在APL中的应用提供了理论依据。有研究表明，基于维奈克拉的治疗可以克服APL对ATO和ATRA的耐药性，提高缓解率，并改善长期生存预后[81]。对于伴FLT3突变的复发难治APL，FLT3抑制剂（如吉瑞替尼）显示出良好前景[82]–[83]。

口服砷剂的应用减少住院需求，降低治疗成本，使APL治疗在资源受限地区更具可行性。未来应进一步评估口服砷剂在不同人群中的疗效和安全性，并优化推广策略，使更多患者受益。

APL的治愈之路是肿瘤精准治疗的典范。从ATRA和ATO的发现，到无化疗协同靶向治疗策略的优化，再到口服砷剂的应用推广，中国学者在每一次突破中均作出了重要贡献。未来，仍需深入研究耐药机制，优化个体化治疗方案，早期诊断、早期治疗，显著降低早期死亡率，并推动新型靶向药物的开发，以最终实现APL的人人有治愈的机遇。

## References

[1] Wang ZY, Chen Z (2008). Acute promyelocytic leukemia: from highly fatal to highly curable[J]. Blood.

[2] Sanz MA, Barragán E (2021). History of acute promyelocytic leukemia[J]. Clin Hematol Int.

[3] Xu ZL, Huang XJ (2020). Therapeutic approaches for acute promyelocytic leukaemia: moving towards an orally chemotherapy-free era[J]. Front Oncol.

[4] Bernard J, Weil M, Boiron M (1973). Acute promyelocytic leukemia: results of treatment by daunorubicin[J]. Blood.

[5] Breitman TR, Selonick SE, Collins SJ (1980). Induction of differentiation of the human promyelocytic leukemia cell line (HL-60) by retinoic acid[J]. Proc Natl Acad Sci U S A.

[6] Breitman TR, Collins SJ, Keene BR (1981). Terminal differentiation of human promyelocytic leukemic cells in primary culture in response to retinoic acid[J]. Blood.

[7] Huang ME, Ye YC, Chen SR (1988). Use of all-trans retinoic acid in the treatment of acute promyelocytic leukemia[J]. Blood.

[8] Chen Z, Chen SJ, Tong JH (1991). The retinoic acid alpha receptor gene is frequently disrupted in its 5′ part in Chinese patients with acute promyelocytic leukemia[J]. Leukemia.

[9] Chen SJ, Zhu YJ, Tong JH (1991). Rearrangements in the second intron of the RARA gene are present in a large majority of patients with acute promyelocytic leukemia and are used as molecular marker for retinoic acid-induced leukemic cell differentiation[J]. Blood.

[10] Chen Z, Brand NJ, Chen A (1993). Fusion between a novel Krüppel-like zinc finger gene and the retinoic acid receptor-alpha locus due to a variant t(11;17) translocation associated with acute promyelocytic leukaemia[J]. EMBO J.

[11] Chen SJ, Zelent A, Tong JH (1993). Rearrangements of the retinoic acid receptor alpha and promyelocytic leukemia zinc finger genes resulting from t(11;17)(q23;q21) in a patient with acute promyelocytic leukemia[J]. J Clin Invest.

[12] Cheng GX, Zhu XH, Men XQ (1999). Distinct leukemia phenotypes in transgenic mice and different corepressor interactions generated by promyelocytic leukemia variant fusion genes PLZF-RARalpha and NPM-RARalpha[J]. Proc Natl Acad Sci U S A.

[13] Chen Z, Guidez F, Rousselot P (1994). PLZF-RAR alpha fusion proteins generated from the variant t(11;17)(q23;q21) translocation in acute promyelocytic leukemia inhibit ligand-dependent transactivation of wild-type retinoic acid receptors[J]. Proc Natl Acad Sci U S A.

[14] Dong S, Zhu J, Reid A (1996). Amino-terminal protein-protein interaction motif (POZ-domain) is responsible for activities of the promyelocytic leukemia zinc finger-retinoic acid receptor-alpha fusion protein[J]. Proc Natl Acad Sci U S A.

[15] Yu M, Tong JH, Mao M (1997). Cloning of a gene (RIG-G) associated with retinoic acid-induced differentiation of acute promyelocytic leukemia cells and representing a new member of a family of interferon-stimulated genes[J]. Proc Natl Acad Sci U S A.

[16] Liu TX, Zhang JW, Tao J (2000). Gene expression networks underlying retinoic acid-induced differentiation of acute promyelocytic leukemia cells[J]. Blood.

[17] Li XY, Jiang LJ, Chen L (2014). RIG-I modulates Src-mediated AKT activation to restrain leukemic stemness[J]. Mol Cell.

[18] Tan Y, Wang X, Song H (2021). A PML/RARα direct target atlas redefines transcriptional deregulation in acute promyelocytic leukemia[J]. Blood.

[19] Muindi J, Frankel SR, Miller WH (1992). Continuous treatment with all-trans retinoic acid causes a progressive reduction in plasma drug concentrations: implications for relapse and retinoid “resistance” in patients with acute promyelocytic leukemia[J]. Blood.

[20] Frankel SR, Eardley A, Heller G (1994). All-trans retinoic acid for acute promyelocytic leukemia. Results of the New York Study[J]. Ann Intern Med.

[21] 全国全反式维甲酸治疗白血病协作组 (1992). 全反式维甲酸治疗544例急性早幼粒细胞白血病的临床研究[J]. 中华血液学杂志.

[22] Fenaux P, Le Deley MC, Castaigne S (1993). Effect of all transretinoic acid in newly diagnosed acute promyelocytic leukemia. Results of a multicenter randomized trial. European APL 91 Group[J]. Blood.

[23] Tallman MS, Andersen JW, Schiffer CA (2002). All-trans retinoic acid in acute promyelocytic leukemia: long-term outcome and prognostic factor analysis from the North American Intergroup protocol[J]. Blood.

[24] Shen ZX, Shi ZZ, Fang J (2004). All-trans retinoic acid/As2O3 combination yields a high quality remission and survival in newly diagnosed acute promyelocytic leukemia[J]. Proc Natl Acad Sci U S A.

[25] 孙 鸿德, 马 玲, 胡 晓晨 (1992). 癌灵Ⅰ号结合中医辨证治疗急性早幼粒细胞白血病32例[J]. 中国中西医结合杂志.

[26] 张 鹏, 王 树叶, 胡 龙虎 (1996). 三氧化二砷注射液治疗72例急性早幼粒细胞白血病[J]. 中华血液学杂志.

[27] Shen ZX, Chen GQ, Ni JH (1997). Use of arsenic trioxide (As2O3) in the treatment of acute promyelocytic leukemia (APL): II. Clinical efficacy and pharmacokinetics in relapsed patients[J]. Blood.

[28] Niu C, Yan H, Yu T (1999). Studies on treatment of acute promyelocytic leukemia with arsenic trioxide: remission induction, follow-up, and molecular monitoring in 11 newly diagnosed and 47 relapsed acute promyelocytic leukemia patients[J]. Blood.

[29] Soignet SL, Frankel SR, Douer D (2001). United States multicenter study of arsenic trioxide in relapsed acute promyelocytic leukemia[J]. J Clin Oncol.

[30] Shigeno K, Naito K, Sahara N (2005). Arsenic trioxide therapy in relapsed or refractory Japanese patients with acute promyelocytic leukemia: updated outcomes of the phase II study and postremission therapies[J]. Int J Hematol.

[31] Mathews V, George B, Chendamarai E (2010). Single-agent arsenic trioxide in the treatment of newly diagnosed acute promyelocytic leukemia: long-term follow-up data[J]. J Clin Oncol.

[32] Ghavamzadeh A, Alimoghaddam K, Rostami S (2011). Phase II study of single-agent arsenic trioxide for the front-line therapy of acute promyelocytic leukemia[J]. J Clin Oncol.

[33] Chen GQ, Shi XG, Tang W (1997). Use of arsenic trioxide (As2O3) in the treatment of acute promyelocytic leukemia (APL): I. As2O3 exerts dose-dependent dual effects on APL cells[J]. Blood.

[34] Chen GQ, Zhu J, Shi XG (1996). In vitro studies on cellular and molecular mechanisms of arsenic trioxide (As2O3) in the treatment of acute promyelocytic leukemia: As2O3 induces NB4 cell apoptosis with downregulation of Bcl-2 expression and modulation of PML-RAR alpha/PML proteins[J]. Blood.

[35] Zhu J, Koken MH, Quignon F (1997). Arsenic-induced PML targeting onto nuclear bodies: implications for the treatment of acute promyelocytic leukemia[J]. Proc Natl Acad Sci U S A.

[36] Zhang XW, Yan XJ, Zhou ZR (2010). Arsenic trioxide controls the fate of the PML-RARalpha oncoprotein by directly binding PML[J]. Science.

[37] Nasr R, Guillemin MC, Ferhi O (2008). Eradication of acute promyelocytic leukemia-initiating cells through PML-RARA degradation[J]. Nat Med.

[38] Zheng PZ, Wang KK, Zhang QY (2005). Systems analysis of transcriptome and proteome in retinoic acid/arsenic trioxide-induced cell differentiation/apoptosis of promyelocytic leukemia[J]. Proc Natl Acad Sci U S A.

[39] Lallemand-Breitenbach V, Guillemin MC, Janin A (1999). Retinoic acid and arsenic synergize to eradicate leukemic cells in a mouse model of acute promyelocytic leukemia[J]. J Exp Med.

[40] Jing Y, Wang L, Xia L (2001). Combined effect of all-trans retinoic acid and arsenic trioxide in acute promyelocytic leukemia cells in vitro and in vivo[J]. Blood.

[41] Hu J, Liu YF, Wu CF (2009). Long-term efficacy and safety of all-trans retinoic acid/arsenic trioxide-based therapy in newly diagnosed acute promyelocytic leukemia[J]. Proc Natl Acad Sci U S A.

[42] Zhu H, Hu J, Chen L (2016). The 12-year follow-up of survival, chronic adverse effects, and retention of arsenic in patients with acute promyelocytic leukemia[J]. Blood.

[43] Thomas X (2019). Acute Promyelocytic Leukemia: A History over 60 Years-From the Most Malignant to the most Curable Form of Acute Leukemia[J]. Oncol Ther.

[44] Sanz MA, Lo Coco F, Martín G (2000). Definition of relapse risk and role of nonanthracycline drugs for consolidation in patients with acute promyelocytic leukemia: a joint study of the PETHEMA and GIMEMA cooperative groups[J]. Blood.

[45] Sanz MA, Montesinos P, Vellenga E (2008). Risk-adapted treatment of acute promyelocytic leukemia with all-trans retinoic acid and anthracycline monochemotherapy: long-term outcome of the LPA 99 multicenter study by the PETHEMA Group[J]. Blood.

[46] Sanz MA, Montesinos P, Rayón C (2010). Risk-adapted treatment of acute promyelocytic leukemia based on all-trans retinoic acid and anthracycline with addition of cytarabine in consolidation therapy for high-risk patients: further improvements in treatment outcome[J]. Blood.

[47] Lo-Coco F, Avvisati G, Vignetti M (2010). Front-line treatment of acute promyelocytic leukemia with AIDA induction followed by risk-adapted consolidation for adults younger than 61 years: results of the AIDA-2000 trial of the GIMEMA Group[J]. Blood.

[48] Iland HJ, Bradstock K, Supple SG (2012). All-trans-retinoic acid, idarubicin, and IV arsenic trioxide as initial therapy in acute promyelocytic leukemia (APML4)[J]. Blood.

[49] Iland HJ, Collins M, Bradstock K (2015). Use of arsenic trioxide in remission induction and consolidation therapy for acute promyelocytic leukaemia in the Australasian Leukaemia and Lymphoma Group (ALLG) APML4 study: a non-randomised phase 2 trial[J]. Lancet Haematol.

[50] Abaza Y, Kantarjian H, Garcia-Manero G (2017). Long-term outcome of acute promyelocytic leukemia treated with all-trans-retinoic acid, arsenic trioxide, and gemtuzumab[J]. Blood.

[51] Lo-Coco F, Avvisati G, Vignetti M (2013). Retinoic acid and arsenic trioxide for acute promyelocytic leukemia[J]. N Engl J Med.

[52] Platzbecker U, Avvisati G, Cicconi L (2017). Improved outcomes with retinoic acid and arsenic trioxide compared with retinoic acid and chemotherapy in non-high-risk acute promyelocytic leukemia: final results of the randomized italian-german APL0406 trial[J]. J Clin Oncol.

[53] Platzbecker U, Adès L, Montesinos P (2024). First results of the APOLLO trial: a randomized phase III study to compare ATO combined with ATRA versus standard AIDA regimen for patients with newly diagnosed, high-risk acute promyelocytic leukemia. EHA.

[54] Voso MT, Guarnera L, Lehmann S (2025). Acute promyelocytic leukemia: long-term outcomes from the HARMONY project[J]. Blood.

[55] Gill H, Raghupathy R, Hou HA (2025). Acute Promyelocytic Leukemia Asian Consortium study of arsenic trioxide in newly diagnosed patients: impact and outcome[J]. Blood Adv.

[56] Chen L, Zhu HM, Li Y (2021). Arsenic trioxide replacing or reducing chemotherapy in consolidation therapy for acute promyelocytic leukemia (APL2012 trial)[J]. Proc Natl Acad Sci U S A.

[57] Dong FY, Zhu HM, Jin W (2024). Chemotherapy-free consolidation is equally effective as chemotherapy-inclusive approaches in high-risk APL patients' post-remission induction. EHA.

[58] Wang HY, Gong S, Li GH (2022). An effective and chemotherapy-free strategy of all-trans retinoic acid and arsenic trioxide for acute promyelocytic leukemia in all risk groups (APL15 trial)[J]. Blood Cancer J.

[59] 中华医学会血液学分会, 中国医师协会血液科医师分会 (2018). 中国急性早幼粒细胞白血病诊疗指南(2018年版)[J]. 中华血液学杂志.

[60] Lu DP, Qiu JY, Jiang B (2002). Tetra-arsenic tetra-sulfide for the treatment of acute promyelocytic leukemia: a pilot report[J]. Blood.

[61] 黄 世林, 郭 爱霞, 向 阳 (1995). 复方青黛片为主治疗急性早幼粒细胞白血病的临床研究[J]. 中华血液学杂志.

[62] 复方黄黛片Ⅱ期临床试验协作组 (2006). 复方黄黛片治疗初诊急性早幼粒细胞白血病的Ⅱ期临床试验[J]. 中华血液学杂志.

[63] Wang L, Zhou GB, Liu P (2008). Dissection of mechanisms of Chinese medicinal formula Realgar-Indigo naturalis as an effective treatment for promyelocytic leukemia[J]. Proc Natl Acad Sci U S A.

[64] Zhu HH, Wu DP, Jin J (2013). Oral tetra-arsenic tetra-sulfide formula versus intravenous arsenic trioxide as first-line treatment of acute promyelocytic leukemia: a multicenter randomized controlled trial[J]. J Clin Oncol.

[65] Zhu HH, Wu DP, Jin J (2016). Long-term survival of acute promyelocytic leukaemia patients treated with arsenic and retinoic acid[J]. Br J Haematol.

[66] Zhu HH, Wu DP, Du X (2018). Oral arsenic plus retinoic acid versus intravenous arsenic plus retinoic acid for non-high-risk acute promyelocytic leukaemia: a non-inferiority, randomised phase 3 trial[J]. Lancet Oncol.

[67] Zhu HH, Liu YR, Jia JS (2018). Oral arsenic and all-trans retinoic acid for high-risk acute promyelocytic leukemia[J]. Blood.

[68] Zhu HH, Hu J, Lo-Coco F (2019). The simpler, the better: oral arsenic for acute promyelocytic leukemia[J]. Blood.

[69] Kumana CR, Mak R, Kwong YL (2020). Resurrection of oral arsenic trioxide for treating acute promyelocytic leukaemia: a historical account from bedside to bench to bedside[J]. Front Oncol.

[70] Gill H, Yim R, Lee H (2018). Long-term outcome of relapsed acute promyelocytic leukemia treated with oral arsenic trioxide-based reinduction and maintenance regimens: A 15-year prospective study[J]. Cancer.

[71] Gill HS, Yim R, Kumana CR (2020). Oral arsenic trioxide, all-trans retinoic acid, and ascorbic acid maintenance after first complete remission in acute promyelocytic leukemia: Long-term results and unique prognostic indicators[J]. Cancer.

[72] Gill H, Kumana CR, Yim R (2019). Oral arsenic trioxide incorporation into frontline treatment with all-trans retinoic acid and chemotherapy in newly diagnosed acute promyelocytic leukemia: A 5-year prospective study[J]. Cancer.

[73] Iland HJ, Reynolds J, Boddy AV (2025). ALLG APML5: bioavailability and safety of oral arsenic trioxide assessed during consolidation therapy for APL[J]. Blood Adv.

[74] Ravandi F, Koumenis I, Johri A (2020). Oral arsenic trioxide ORH-2014 pharmacokinetic and safety profile in patients with advanced hematologic disorders[J]. Haematologica.

[75] Chen SJ, Chen Z, Chen A (1992). Occurrence of distinct PML-RAR-alpha fusion gene isoforms in patients with acute promyelocytic leukemia detected by reverse transcriptase/polymerase chain reaction[J]. Oncogene.

[76] Sanz MA, Fenaux P, Tallman MS (2019). Management of acute promyelocytic leukemia: updated recommendations from an expert panel of the European LeukemiaNet[J]. Blood.

[77] Grimwade D, Jovanovic JV, Hills RK (2009). Prospective minimal residual disease monitoring to predict relapse of acute promyelocytic leukemia and to direct pre-emptive arsenic trioxide therapy[J]. J Clin Oncol.

[78] Schuurhuis GJ, Heuser M, Freeman S (2018). Minimal/measurable residual disease in AML: a consensus document from the European LeukemiaNet MRD Working Party[J]. Blood.

[79] Lo-Coco F, Cimino G, Breccia M (2004). Gemtuzumab ozogamicin (Mylotarg) as a single agent for molecularly relapsed acute promyelocytic leukemia[J]. Blood.

[80] Jen WY, Marvin-Peek J, Kantarjian HM (2025). Long-term follow-up of a phase 2 study of all-trans retinoic acid, arsenic trioxide, and gemtuzumab ozogamicin in acute promyelocytic leukemia[J]. Cancer.

[81] Wang QQ, Wang HF, Zhao JZ (2022). Venetoclax for arsenic-resistant acute promyelocytic leukaemia[J]. Br J Haematol.

[82] Kobayashi H, Tsutsumi H, Misaki Y (2023). Gilteritinib monotherapy as a transplant bridging option for a patient with FLT3-mutated acute promyelocytic leukemia who developed a second relapse after all-trans retinoic acid + chemotherapy, arsenic trioxide, and high-dose cytarabine therapy[J]. Case Rep Hematol.

[83] Hou CX, Chen Y, Liu SH (2024). Effective treatment with Gilteritinib-based regimens for FLT3-mutant extramedullary relapse in acute promyelocytic leukemia[J]. Hematology.

